# A phase 1, randomized, double‐blind, placebo‐controlled study to evaluate the safety, tolerability, and pharmacokinetics of single and multiple doses of lisdexamfetamine dimesylate in Japanese and Caucasian healthy adult subjects

**DOI:** 10.1002/npr2.12082

**Published:** 2019-11-25

**Authors:** James Ermer, Patrick Martin, Mary Corcoran, Yumiko Matsuo

**Affiliations:** ^1^ Shire Wayne PA USA; ^2^ Shire Lexington MA USA; ^3^ Shionogi & Co., Ltd Osaka Japan; ^4^ Present address: Pharmacology Development Service Senior Independent Consultant Eagleville PA USA

**Keywords:** attention‐deficit/hyperactivity disorder, d*‐*amphetamine, lisdexamfetamine dimesylate, pharmacokinetics, psychopharmacology: clinical, safety

## Abstract

**Aim:**

To assess safety, tolerability, and pharmacokinetics of lisdexamfetamine dimesylate in Japanese and Caucasian healthy adults.

**Methods:**

A phase 1, double‐blind, randomized, placebo‐controlled, single‐ and multiple‐dose study in Japanese and Caucasian subjects. Subjects received lisdexamfetamine 20 mg or placebo on Day 1, then lisdexamfetamine 20 mg/d (Days 4‐8), 50 mg/d (Days 9‐13), 70 mg/d (Days 14‐18), or matching placebo. Pharmacokinetic parameters for lisdexamfetamine and d *‐*amphetamine were estimated by noncompartmental analysis.

**Results:**

Fifteen Japanese and 19 Caucasian subjects were enrolled and randomized. The lisdexamfetamine and d *‐*amphetamine plasma concentration‐time curves were similar for both ethnic groups following single and multiple doses. Mean area under the concentration‐time curves for d *‐*amphetamine were higher (by 11%‐15%) in Japanese than Caucasian subjects following multiple dosing of lisdexamfetamine. Mean bodyweight was 17% lower in Japanese than Caucasian subjects. Weight‐corrected means for oral clearance were similar in both ethnic groups, with no unexpected accumulation of d *‐*amphetamine. Lisdexamfetamine was generally well tolerated by both ethnic groups, with no serious adverse events reported. The 10/12 Japanese and 11/16 Caucasian subjects who received lisdexamfetamine completed the study; two Japanese and three Caucasian subjects discontinued due to adverse events. Most adverse events were of mild severity.

**Conclusion:**

Pharmacokinetics were generally similar for Japanese and Caucasian subjects; the minor differences observed were likely due to bodyweight differences in the two ethnic groups. Lisdexamfetamine was generally well tolerated. Adverse events were consistent with the established safety profile of lisdexamfetamine and were similar in both ethnic groups.

## Abstract 2

This phase 1, double‐blind, randomized, placebo‐controlled study was conducted to assess safety, tolerability, and pharmacokinetics of lisdexamfetamine dimesylate in Japanese and Caucasian subjects. Fifteen Japanese and 19 Caucasian subjects were enrolled and randomized to receive lisdexamfetamine 20 mg (Day 1), 20 mg/d (Days 4‐8), 50 mg/d (Days 9‐13), and 70 mg/d (Days 14‐18), or matching placebo. Pharmacokinetics were generally similar for Japanese and Caucasian subjects; minor differences observed were likely due to bodyweight differences in the two ethnic groups, and lisdexamfetamine was generally well tolerated.

## Graphical abstract

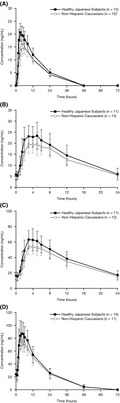

## 1 INTRODUCTION

Lisdexamfetamine dimesylate, the first prodrug stimulant indicated for the treatment of attention‐deficit/hyperactivity disorder (ADHD), was developed to provide a consistent and extended effect, with less potential for abuse and reduced overdose toxicity.^1^ Lisdexamfetamine is available for the treatment of ADHD in several countries, including the USA and Canada in children, adolescents, and adults (Vyvanse^®^; Shire LLC), and in the EU in children (Elvanse^®^; Shire LLC).^2^

After oral ingestion, lisdexamfetamine (the therapeutically inactive molecule) is converted to l‐lysine and therapeutically active d *‐*amphetamine.^3^ The pharmacokinetics of lisdexamfetamine has been studied in healthy subjects and patients with ADHD.^4^, ^5^ A small study examining single‐dose administration of lisdexamfetamine in children with ADHD found that plasma concentrations of d *‐*amphetamine were dose proportional with low interpatient variability.^4^ Dose proportionality and predictability of pharmacokinetic properties over a range of lisdexamfetamine doses have been described in a study of healthy adults.^5^ Furthermore, after a single 70 mg oral dose of lisdexamfetamine, pharmacokinetic analysis showed that it extensively metabolized to d *‐*amphetamine and its derivatives before rapid elimination.^6^ Lisdexamfetamine has been reported to have a half‐life of <1 hour.^6^ In a study in healthy adults, systemic exposure to d *‐*amphetamine was around 20 times higher than that of intact lisdexamfetamine.^6^ In addition, d *‐*amphetamine or lisdexamfetamine bioavailability in healthy adults does not appear to be significantly affected by food,^7^ and the pharmacokinetics of lisdexamfetamine or d *‐*amphetamine has been found to be consistent between men and women.^8^

Within 6 hours postadministration, lisdexamfetamine was completely eliminated from subjects in a phase 1 study of oral lisdexamfetamine 70 mg/d in healthy adults.^1^ d *‐*amphetamine achieved a steady state (based on minimum plasma concentration [*C*_min_]) on Day 5; the maximum plasma concentration (*C*_max_; mean ± standard deviation [SD]) at steady state was 90.1 ± 29.6 ng/mL. The median time to *C*_max_ (*T*_max_) for d *‐*amphetamine was 3 hours. Lisdexamfetamine was undetectable on Day 5, while *C*_min_ for d *‐*amphetamine was recorded as 20.6 ng/mL. It was found that 95% of d *‐*amphetamine was absent from plasma samples 48 hours after administration of the last dose on Day 7. The area under the plasma concentration‐time curve (AUC) from time 0 to time infinity (AUC_0‐∞_) for d *‐*amphetamine was 1453 ± 645.7 ng h/mL.^1^

Prior pharmacological treatment had no impact on the dose‐response efficacy of lisdexamfetamine (30‐70 mg/d) in a 4 week, randomized, placebo‐controlled, forced‐dose titration study in adults with ADHD.^9^ In addition, the study found an increasing effect size with increasing lisdexamfetamine dose, and a stronger dose‐response effect for severe baseline hyperactive‐impulsive symptom scores and lisdexamfetamine dose when compared with less severe symptom scores.^9^ Furthermore, short‐ and long‐term studies have shown lisdexamfetamine to be effective and well tolerated in the treatment of ADHD in children,^10^, ^11^, ^12^, ^13^, ^14^, ^15^ adolescents,^14^, ^15^, ^16^ and adults.^17^, ^18^, ^19^, ^20^, ^21^, ^22^, ^23^

The primary objective of the current study was to assess the safety, tolerability, and pharmacokinetics of lisdexamfetamine dimesylate after single and multiple oral doses in healthy adults of Japanese descent. The secondary objectives were to assess these parameters in healthy adults of non‐Hispanic Caucasian descent (referred to as Caucasian hereafter) and to compare the safety and pharmacokinetic profiles of Japanese and Caucasian subjects.

## 2 METHODS

### 2.1 Study design and methods

This was a phase 1, double‐blind, randomized, placebo‐controlled, single‐ and multiple‐dose study to evaluate the safety, tolerability, and pharmacokinetics of lisdexamfetamine dimesylate administered in healthy adult subjects of Japanese or Caucasian descent. The study was conducted at a single site in the USA. The study protocol/protocol amendments, and relevant supporting information/documents, were approved by the Institutional Review Board (IRB) prior to study initiation (Alpha IRB). The study was conducted in accordance with International Conference on Harmonisation of Good Clinical Practice guidelines, the principles of the Declaration of Helsinki, as well as other applicable local ethical and legal requirements. Subjects provided written informed consent.

Subjects were aged 18‐55 years with a body mass index (BMI) 18.0‐30.0 kg/m^2^ inclusive and bodyweight 50 kg or more. The main exclusion criteria included are as follows: suicide risk; history of hypertension; history of seizure (other than infantile febrile seizures); any tic disorder or current diagnosis and/or family history of Tourette's syndrome; history of cardiovascular diseases; and glaucoma. Women who were pregnant were excluded from this study, but nonlactating women who were at least 90 days postpartum or nulliparous could participate. All subjects were required to comply with the applicable contraceptive requirements of the protocol. Subjects were also required to be willing and able to consume standardized meals during the confinement period of the study. Eligibility of subjects was confirmed during the screening period (Days −28 to −2) and reconfirmed on Day −1 of treatment. Subjects were enrolled as pairs such that Caucasian subjects matched Japanese subjects based on sex, age (±10 years), and BMI (±15%).

Subjects were confined to the clinical research center during the treatment period after checking in on Day −1, when protocol‐defined safety assessments were made. On Day 1, eligible subjects were randomized (4:1) to receive lisdexamfetamine 20 mg or identical placebo capsules administered orally. Subjects were randomized according to the schedule developed by PRA International through the use of a four‐digit randomization number allocated prior to dosing. Treatment assignments giving details of individual subject treatment were provided in code‐break envelopes, which were held at the site by the designated unblinded pharmacist or dispenser. Placebo and lisdexamfetamine capsules were overencapsulated to ensure both treatments were similar in shape, size, weight, and color.

On Day 4, safety assessments were made and subjects received lisdexamfetamine 20 mg (Days 4‐8), lisdexamfetamine 50 mg (Days 9‐13), lisdexamfetamine 70 mg (Days 14‐18), or placebo once daily. Subjects were discharged from the study center on Day 21 after study‐related assessments were completed. A telephone follow‐up was conducted 7 days (±2 days) after subjects received the last dose of lisdexamfetamine or placebo to record adverse events (AEs) and any changes in concomitant medications.

### 2.2 Number of subjects (planned)

The study was designed to enroll 30 healthy male and female subjects with the intention that in each ethnic group, 12 subjects were to receive single and multiple doses of lisdexamfetamine, and 3 subjects were to receive single and multiple doses of placebo. A minimum of 12 Japanese and 12 Caucasian subjects were required to complete the study. No formal calculations were performed to determine sample size, which was based on feasibility and similar to that of comparable studies.^5^, ^24^

### 2.3 Pharmacokinetic evaluation

Blood sampling for pharmacokinetic analysis was performed at prespecified time points. Plasma samples from subjects dosed with lisdexamfetamine were analyzed for lisdexamfetamine and d *‐*amphetamine using validated liquid chromatography with tandem mass spectrometry detection methods. The method was linear over the range 1‐100 ng/mL for lisdexamfetamine and 2‐200 ng/mL for d *‐*amphetamine, with lower limits of quantification of 1 ng/mL for lisdexamfetamine and 2 ng/mL for d *‐*amphetamine. Plasma quality control samples were prepared in control human plasma at three concentration levels (3, 20, and 80 ng/mL for lisdexamfetamine; and 6, 40, and 160 ng/mL for d *‐*amphetamine), stored with the study samples, and assayed with each batch of study samples against freshly prepared calibration standards. The data for both calibration standards and quality control samples were in accordance with the US Food and Drug Administration Guidance for Industry.^25^

Pharmacokinetic parameters, including *C*_max_, AUC, and apparent oral clearance (CL/F), were determined from the actual blood sampling times and plasma concentration‐time data for lisdexamfetamine and d *‐*amphetamine by noncompartmental analysis.

### 2.4 Safety and tolerability assessments

Subjects were questioned about AEs at screening, each day during each treatment period and during the follow‐up telephone call. AEs were recorded and classified using version 15.1 of the Medical Dictionary for Regulatory Activities, by system organ class (SOC) and preferred term, throughout the study period and for 7 (±2) days after the last dose. An AE (classified by preferred term) was considered a treatment‐emergent AE (TEAE) if it began on or after the first dose of investigational product, or if it began before the first dose but increased in severity on or after the first dose of investigational product. If more than one AE with the same preferred term was reported before the date of the first dose of investigational product, then the AE with the greatest severity was used as the benchmark for comparison to the AEs occurring after the date of first dose under the preferred term. An AE that occurred more than 3 days after the date of the last dose of investigational product was not counted as a TEAE. If more than one AE occurred with the same preferred term for the same subject, then the subject was counted only once for that preferred term using the most severe or most related occurrence for the summarization by severity and by relationship to investigational product.

Other safety assessments included vital signs (systolic blood pressure [SBP], diastolic blood pressure [DBP], and pulse rate) at screening and each day, and electrocardiograms (ECGs), physical examinations, clinical laboratory tests (biochemistry, hematology, and urinalysis), and the Columbia Suicide Severity Rating Scale (C‐SSRS)^26^ at the screening visit and on Days −1, 4, and 21.

### 2.5 Statistical methods

Summary statistics (including means and SDs) were calculated for lisdexamfetamine and d *‐*amphetamine plasma concentrations and for pharmacokinetic parameters, and are presented by dose and ethnic group.

The safety analysis group included all enrolled subjects who took at least one dose of investigational product and had at least one postdose safety assessment. The pharmacokinetic parameter analysis group included all subjects with at least one pharmacokinetic parameter estimated adequately in the pharmacokinetic concentration analysis group (all subjects who took at least one dose of the investigational product and who underwent pharmacokinetic sampling and had evaluable pharmacokinetic assay results for lisdexamfetamine and d *‐*amphetamine).

The number and percentage of subjects with any TEAE, severe TEAE, TEAEs related to the investigational product, serious TEAE, or TEAE leading to discontinuation were calculated. The number and percentage of subjects with any TEAE classified by SOC and preferred term were calculated. Clinical laboratory test parameters (continuous variables) were summarized by treatment arm and ethnic group using summary statistics for the actual value and for change from baseline at each time point. The number and percentage of subjects with potentially clinically important (PCI) postbaseline laboratory values were tabulated by treatment arm and ethnic group. Vital signs and ECG results were summarized by treatment arm and ethnic group using summary statistics for actual values and for change from baseline at each time point. The number and percentage of subjects with PCI postbaseline vital signs and ECG values were tabulated by treatment and ethnic groups. C‐SSRS responses and physical examination results were listed by subject.

## 3 RESULTS

A total of 34 healthy subjects (24 men and 10 women) were enrolled in the study and received at least one dose of investigational product. Of the 34 subjects, 15 were of Japanese descent and 19 were Caucasian. Baseline characteristics were generally similar in both ethnic groups (Table 1). However, although subjects were matched for BMI, Japanese subjects were lighter (mean weight 63.09 vs 75.46 kg) and shorter (mean height 167.10 vs 177.84 cm) than Caucasian subjects.

**Table 1:** Demographic and baseline characteristics for Japanese and Caucasian subjects overall and by treatment group

Characteristic^a^		Placebo		Lisdexamfetamine		Both treatments	
		Japanese (n = 3)	Caucasian (n = 3)	Japanese (n = 12)	Caucasian (n = 16)	Japanese (n = 15)	Caucasian (n = 19)
Age (y)^b^	Mean (SD)	37.0 (15.13)	38.3 (13.43)	39.2 (10.60)	37.1 (11.35)	38.7 (11.04)	37.3 (11.29)
	Median	42.0	44.0	40.5	35.0	41.0	38.0
	Min, max	20, 49	23, 48	23, 52	22, 54	20, 52	22, 54
Sex							
Male	n (%)	2 (66.7)	2 (66.7)	9 (75.0)	11 (68.8)	11 (73.3)	13 (68.4)
Female	n (%)	1 (33.3)	1 (33.3)	3 (25.0)	5 (31.3)	4 (26.7)	6 (31.6)
Weight (kg)	Mean (SD)	66.20 (5.65)	76.97 (2.71)	62.32 (6.36)	75.18 (12.26)	63.09 (6.24)	75.46 (11.25)
	Median	66.30	76.70	64.45	75.85	65.20	76.70
	Min, max	60.5, 71.8	74.4, 79.8	51.2, 70.6	52.7, 91.3	51.2, 71.8	52.7, 91.3
Height (cm)	Mean (SD)	168.23 (4.88)	181.40 (10.96)	166.82 (7.02)	177.18 (9.69)	167.10 (6.51)	177.84 (9.70)
	Median	170.90	185.40	167.30	178.00	168.00	178.80
	Min, max	162.6, 171.2	169.0, 189.8	152.8, 179.1	158.8, 190.4	152.8, 179.1	158.8, 190.4
BMI (kg/m^2^)^c^	Mean (SD)	23.36 (1.06)	23.50 (2.21)	22.41 (2.10)	23.78 (2.02)	22.60 (1.95)	23.73 (1.99)
	Median	22.88	22.31	22.23	23.99	22.62	23.82
	Min, max	22.62, 24.58	22.15, 26.05	19.58, 26.32	20.90, 28.92	19.58, 26.32	20.90, 28.92

Abbreviations: BMI, body mass index; SD, standard deviation.

a The baseline value for a characteristic is the value from the visit time point as specified in the statistical analysis plan.

b Age was calculated as the difference between date of birth and date of informed consent, truncated to years.

c BMI was calculated as weight (kg)/height (m^2^).

### 3.1 Subject disposition

**Figure 1:**
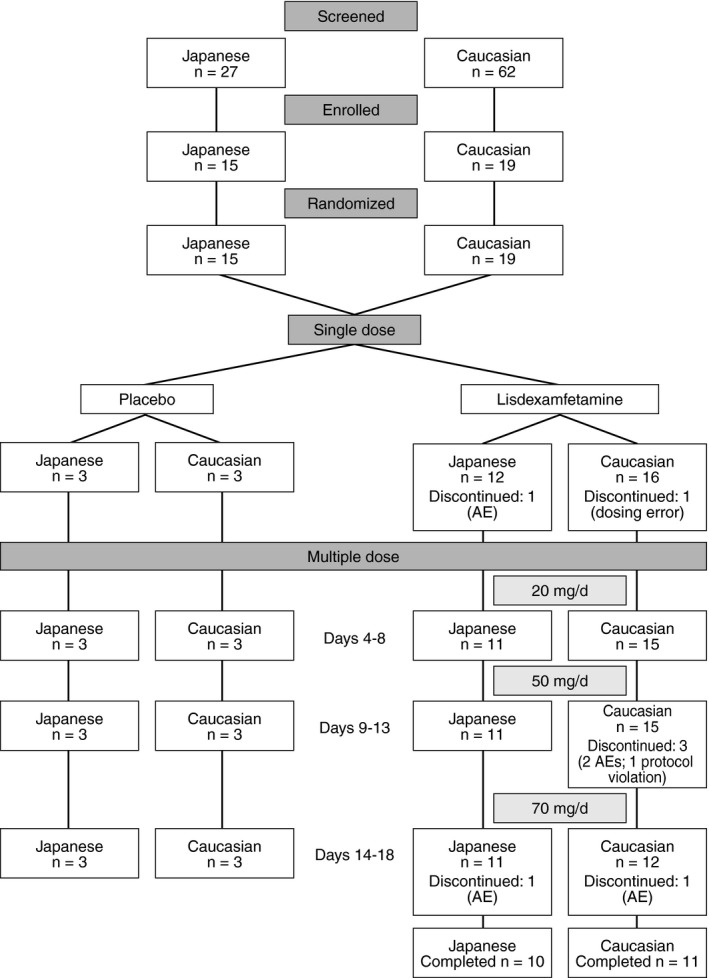
Subject disposition

In the lisdexamfetamine groups, 10/12 Japanese subjects and 11/16 Caucasian subjects completed the study (Figure 1). Two Japanese subjects randomized to receive lisdexamfetamine discontinued the study due to a mild AE (one during the single‐dose period and the other during the multiple‐dose period). Five Caucasian subjects in the lisdexamfetamine group discontinued from the study, three due to AEs that were mild or moderate in severity (see safety results), one due to dosing error, and one due to a protocol violation. All subjects in the placebo groups completed the study.

### 3.2 Pharmacokinetics

**Figure 2:**
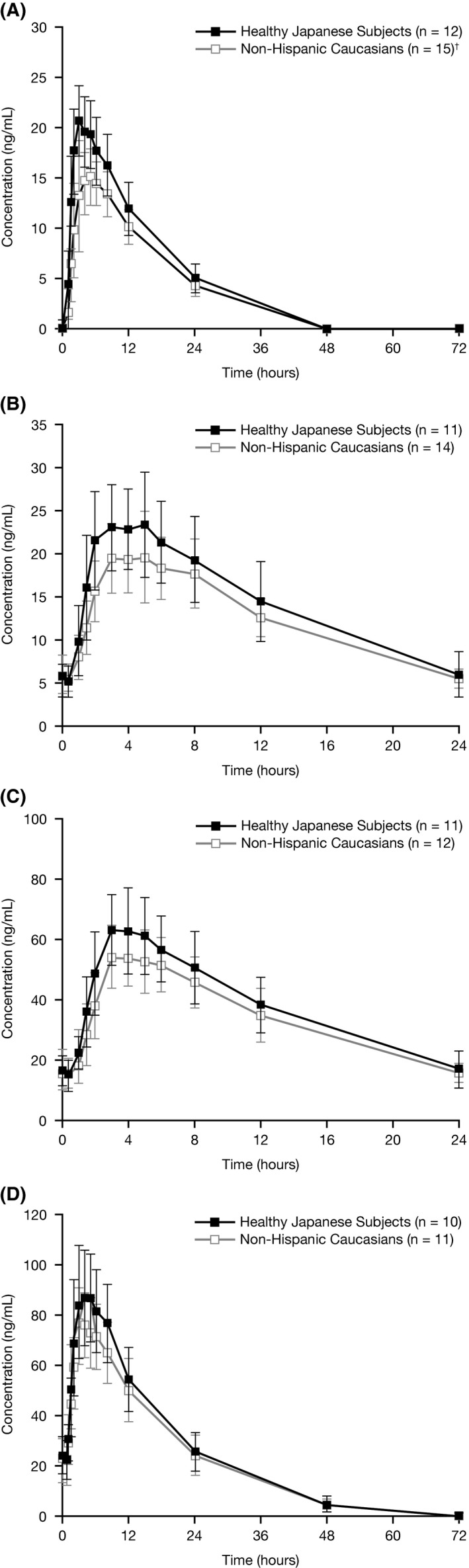
d *‐*amphetamine plasma concentration (mean [SD]) following administration of lisdexamfetamine (A) single 20 mg dose, (B) 20 mg multiple dose, (C) 50 mg multiple dose, and (D) 70 mg multiple dose, by ethnic group. ^†^At time points 0.5, 4‐72 h (n = 14)

For lisdexamfetamine (not shown) and d *‐*amphetamine (Figure 2), the plasma concentration‐time curves were generally similar in shape for Japanese and Caucasian subjects following a single‐dose and at each lisdexamfetamine multiple‐dose level.

Selected plasma pharmacokinetic parameters by dose and ethnic group are shown for lisdexamfetamine in Table 2 and for d *‐*amphetamine in Table 3. Mean *C*_max_ for d *‐*amphetamine was higher (by approximately 34%) in Japanese than Caucasian subjects with single dosing (21.14 vs 15.79 ng/mL, respectively) and at each lisdexamfetamine multiple‐dose level (range approximately 16%‐24% higher for d *‐*amphetamine in Japanese subjects). As a result, mean AUC values from time 0 to the time point of the dosage interval (AUC_τ_) for the multiple‐dose period for d *‐*amphetamine in Japanese subjects were also generally higher (by approximately 11%‐15%) than those seen in Caucasian subjects. Regression analyses of AUC_τ_ and *C*_max_ demonstrated a linear relationship between exposure to d *‐*amphetamine and dose increase; AUC_τ_ slope estimates were 1.07 for Japanese subjects and 1.11 for Caucasian subjects, and slope estimates for *C*_max_ were 1.02 for Japanese subjects and 1.09 for Caucasian subjects.

**Table 2:** Lisdexamfetamine pharmacokinetic parameters in plasma by ethnic group in the single‐ and multiple‐dose periods

Parameter	Single lisdexamfetamine dose		Multiple lisdexamfetamine dose					
	20 mg		20 mg/d		50 mg/d		70 mg/d	
	Japanese (n = 12)	Caucasian (n = 15)	Japanese (n = 11)	Caucasian (n = 14)	Japanese (n = 11)	Caucasian (n = 12)	Japanese (n = 10)	Caucasian (n = 11)
	Mean (SD)		Mean (SD)		Mean (SD)		Mean (SD)	
*C*_max_, ng/mL	8.52 (2.63)	6.32 (2.21)	8.82 (2.44)	7.07 (2.96)	33.58 (10.19)	26.44 (8.84)	47.27 (19.94)	50.79 (23.07)
*t*_½_, h	0.44 (0.01)^b^	0.40 (0.03)^c^	0.40 (0.13)^b^	0.46 (0.07)^d^	0.46 (0.07)	0.50 (0.09)	0.51 (0.09)	0.53 (0.15)
AUC^a^, ng h/mL	12.35 (3.81)^b^	9.30 (1.92)^c^	10.50 (2.69)^b^	9.21 (3.30)^d^	41.32 (10.52)	35.83 (13.04)	65.89 (23.09)	59.14 (23.68)
CL/F, L/h	1715.70 (473.46)^b^	2219.84 (454.15)^c^	1988.41 (489.32)^b^	2465.62 (967.26)^d^	1293.01 (370.73)	1570.13 (575.34)	1246.47 (636.70)	1376.96 (580.77)
Weight‐adjusted CL/F, L/h/kg	28.82 (9.89)^b^	33.12 (7.12)^c^	33.12 (3.71)^b^	33.12 (9.66)^d^	20.43 (4.66)	21.20 (6.28)	19.70 (9.01)	17.84 (6.06)
*T*_max_, median (range), h	1.0 (1‐1.5)	1.0 (1‐4)	1.0 (1‐1.5)	1.0 (0.5‐2)	1.0 (1‐2)	1.5 (1‐2)	1.5 (1‐3)	1.0 (1‐1.5)

Abbreviations: AUC, area under the plasma concentration‐time curve; AUC_0‐∞_, AUC from time 0 to time infinity; AUC_τ_, AUC over the dosing interval τ; CL/F, apparent oral clearance where CL/F = dose/AUC_0‐∞_ for single dose and dose/AUC_τ_ for multiple dose, where F denotes the bioavailability of lisdexamfetamine; *C*_max_, maximum plasma concentration; SD, standard deviation; *t*_½_, terminal elimination half‐life where *t*_½_ = (ln_2_)/λ_z_; *T*_max_, time to *C*_max_.

a For single dose, AUC_0‐∞_; for multiple dose, AUC_τ_.

b n = 3.

c n = 4.

d n = 8.

**Table 3:** d *‐*amphetamine pharmacokinetic parameters in plasma by ethnic group in the single‐ and multiple‐dose periods

Parameter	Single lisdexamfetamine dose		Multiple lisdexamfetamine dose					
	20 mg		20 mg/d		50 mg/d		70 mg/d	
	Japanese (n = 12)	Caucasian (n = 15)	Japanese (n = 11)	Caucasian (n = 14)	Japanese (n = 11)	Caucasian (n = 12)	Japanese (n = 10)	Caucasian (n = 11)
	Mean (SD)		Mean (SD)		Mean (SD)		Mean (SD)	
*C*_max_, ng/mL	21.14 (3.32)	15.79 (2.79)	25.80 (5.29)	20.76 (4.51)	66.12 (13.24)	56.86 (9.58)	92.07 (16.51)	79.20 (13.43)
*t*_½_, h	9.65 (1.48)	10.11 (1.96)^b^	9.38 (1.88)	9.72 (1.49)	10.28 (2.10)	10.27 (1.92)	9.71 (1.41)	10.76 (1.65)
AUC^a^, ng h/mL	348.83 (74.37)	282.77 (45.64)^b^	335.84 (89.73)	291.78 (48.31)	889.48 (191.83)	793.24 (162.24)	1280.56 (290.06)	1153.45 (241.44)
CL/F, L/h	59.46 (11.02)	72.56 (12.40)^b^	62.98 (14.56)	70.05 (9.89)	58.41 (11.36)	65.40 (12.85)	57.06 (11.95)	62.89 (11.99)
Weight‐adjusted CL/F, L/h/kg	0.95 (0.15)	1.00 (0.27)^b^	1.00 (0.19)	0.95 (0.21)	0.93 (0.15)	0.91 (0.28)	0.91 (0.16)	0.85 (0.23)
*T*_max_, median (range), h	3.0 (2‐4)	4.0 (2‐6)	3.0 (1.5‐5)	4.5 (3‐8.1)	4.0 (3‐5)	3.5 (3‐6)	5.0 (3‐8)	4.0 (3‐6)

Abbreviations: AUC, area under the plasma concentration‐time curve; AUC_0‐∞_, AUC from time 0 to time infinity; AUC_τ_, AUC over the dosing interval τ; CL/F, apparent oral clearance calculated as dose of lisdexamfetamine/AUC_0‐∞_ of d *‐*amphetamine for single dose and as dose of lisdexamfetamine/AUC_τ_ of d *‐*amphetamine for multiple dose, where F denotes the combined parameter of the fraction of an administered lisdexamfetamine dose which reached the systemic circulation and the fraction which was converted to d *‐*amphetamine; *C*_max_, maximum plasma concentration; SD, standard deviation; *t*_½_, terminal elimination half‐life where *t*_½_ = (ln_2_)/λ_z_; *T*_max_, time to *C*_max_.

a For single dose, AUC_0‐∞_; for multiple dose, AUC_τ_.

b n = 14.

The median *T*_max_ and mean terminal elimination half‐life (*t*_½_) of lisdexamfetamine were similar in both ethnic groups across both treatment periods (1.0‐1.5 and 0.40‐0.53 hours, respectively). For d *‐*amphetamine, the median *T*_max_ occurred 3‐5 hours after dosing, and mean *t*_½_ was around 10 hours for both ethnic groups in both treatment periods. The mean AUC_τ_ and *C*_max_ for d *‐*amphetamine behaved in a linear, dose‐proportional manner across the dose range studied (20‐70 mg) for both Japanese and Caucasian subjects (Table 3).

The weight‐corrected total or CL/F and *t*_½_ for d *‐*amphetamine were generally consistent across the dose range studied and were similar in both subject groups (Table 3). The mean apparent terminal phase volume of distribution (*V*_z_/*F*) showed similar characteristics in both ethnic groups: 13.1 and 13.9 L/kg for Japanese and Caucasian subjects, respectively. The means for AUC on Day 8/Day 1 (*R*_AUC_) and for *C*_max_ on Day 8/Day 1 (RC_max_) show that there was no unexpected accumulation of d *‐*amphetamine in either ethnic group. For d *‐*amphetamine, R_AUC_ was 1.19 for Japanese subjects and 1.34 for Caucasian subjects; RC_max_ was 1.21 for Japanese subjects and 1.29 for Caucasian subjects.

The means for CL/F, weight‐corrected CL/F, and *t*_½_ for d *‐*amphetamine were similar for all pairwise dose comparisons (ie, lisdexamfetamine 50/20, 70/20, and 70/50 mg) and each parameter in both ethnic groups.

### 3.3 Safety results

Single (20 mg) and multiple (20, 50, and 70 mg/d) oral doses of lisdexamfetamine were generally well tolerated by Japanese and Caucasian subjects. During the single‐dose period in the lisdexamfetamine groups, 33.3% and 6.3% of Japanese and Caucasian subjects, respectively, experienced at least one TEAE (all considered mild). During the same dosing period, 33.3% and 0% of Japanese and Caucasian subjects, respectively, experienced TEAEs in the placebo group. The most common TEAEs during the multiple‐dose period are shown in Table 4. During the multiple‐dose period in the lisdexamfetamine groups (all doses), 72.7% and 80.0% of Japanese and Caucasian subjects, respectively, experienced at least one TEAE; all were considered to be mild with the exception of two classed as moderate (dizziness by one Caucasian subject receiving 20 mg and palpitations by one Japanese subject receiving 20 mg). In both ethnic groups, the recorded TEAEs were highest during the 50 mg dose. A Japanese subject experienced at least one TEAE in the placebo group. There were no serious or severe TEAEs during the study in either ethnic group.

**Table 4:** TEAEs by ethnic group during the multiple‐dose period

TEAE n (%) m	Placebo		Multiple lisdexamfetamine dose							
			20 mg		50 mg		70 mg		All doses	
	Japanese (n = 3)	Caucasian (n = 3)	Japanese (n = 11)	Caucasian (n = 15)	Japanese (n = 11)	Caucasian (n = 15)	Japanese (n = 11)	Caucasian (n = 12)	Japanese (n = 11)	Caucasian (n = 15)
Any TEAE	1 (33.3) 2	0	4 (36.4) 9	6 (40.0) 7	5 (45.5) 8	9 (60.0) 19	3 (27.3) 9	4 (33.3) 5	8 (72.7) 26	12 (80.0) 31
Gastrointestinal disorders	0	0	0	0	0	1 (6.7) 1	2 (18.2) 2	1 (8.3) 1	2 (18.2) 2	2 (13.3) 2
Constipation	0	0	0	0	0	1 (6.7) 1	0	0	0	1 (6.7) 1
Diarrhea	0	0	0	0	0	0	1 (9.1) 1	1 (8.3) 1	1 (9.1) 1	1 (6.7) 1
Nausea	0	0	0	0	0	0	1 (9.1) 1	0	1 (9.1) 1	0
General disorders and administration site conditions	1 (33.3) 1	0	0	0	1 (9.1) 1	2 (13.3) 3	0	0	1 (9.1) 1	2 (13.3) 3
Chest discomfort	0	0	0	0	0	1 (6.7) 1	0	0	0	1 (6.7) 1
Fatigue	0	0	0	0	0	1 (6.7) 1	0	0	0	1 (6.7) 1
Feeling abnormal	1 (33.3) 1	0	0	0	0	0	0	0	0	0
Pain	0	0	0	0	0	1 (6.7) 1	0	0	0	1 (6.7) 1
Thirst	0	0	0	0	1 (9.1) 1	0	0	0	1 (9.1) 1	0
Metabolism and nutrition disorders	0	0	1 (9.1) 1	1 (6.7) 1	0	1 (6.7) 1	1 (9.1) 1	0	2 (18.2) 2	2 (13.3) 2
Decreased appetite	0	0	1 (9.1) 1	1 (6.7) 1	0	1 (6.7) 1	0	0	1 (9.1) 1	2 (13.3) 2
Dehydration	0	0	0	0	0	0	1 (9.1) 1	0	1 (9.1) 1	0
Musculoskeletal and connective tissue disorders	0	0	0	0	1 (9.1) 1	0	1 (9.1) 1	0	1 (9.1) 2	0
Musculoskeletal discomfort	0	0	0	0	1 (9.1) 1	0	0	0	1 (9.1) 1	0
Neck pain	0	0	0	0	0	0	1 (9.1) 1	0	1 (9.1) 1	0
Nervous system disorders	0	0	3 (27.3) 4	2 (13.3) 3	1 (9.1) 1	4 (26.7) 5	2 (18.2) 3	1 (8.3) 1	5 (45.5) 8	5 (33.3) 9
Dizziness	0	0	1 (9.1) 1	0	0	1 (6.7) 1	1 (9.1) 1	1 (8.3) 1	2 (18.2) 2	2 (13.3) 2
Headache	0	0	2 (18.2) 2	1 (6.7) 1	0	4 (26.7) 4	1 (9.1) 1	0	3 (27.3) 3	4 (26.7) 5
Hypersomnia	0	0	0	0	0	0	1 (9.1) 1	0	1 (9.1) 1	0
Hypoesthesia	0	0	1 (9.1) 1	0	0	0	0	0	1 (9.1) 1	0
Paresthesia	0	0	0	0	1 (9.1) 1	0	0	0	1 (9.1) 1	0
Presyncope	0	0	0	1 (6.7) 2	0	0	0	0	0	1 (6.7) 2
Psychiatric disorders	0	0	1 (9.1) 1	2 (13.3) 2	1 (9.1) 2	5 (33.3) 7	1 (9.1) 1	3 (25.0) 3	2 (18.2) 4	8 (53.3) 12
Abnormal dreams	0	0	0	0	0	0	1 (9.1) 1	0	1 (9.1) 1	0
Anxiety	0	0	1 (9.1) 1	0	0	2 (13.3) 2	0	2 (16.7) 2	1 (9.1) 1	3 (20.0) 4
Apathy	0	0	0	0	0	1 (6.7) 1	0	0	0	1 (6.7) 1
Dysphoria	0	0	0	0	0	1 (6.7) 1	0	0	0	1 (6.7) 1
Euphoric mood	0	0	0	0	0	1 (6.7) 1	0	0	0	1 (6.7) 1
Insomnia	0	0	0	1 (6.7) 1	0	2 (13.3) 2	0	0	0	3 (20.0) 3
Mood altered	0	0	0	0	1 (9.1) 1	0	0	0	1 (9.1) 1	0
Mood swings	0	0	0	0	1 (9.1) 1	0	0	0	1 (9.1) 1	0
Sleep disorder	0	0	0	1 (6.7) 1	0	0	0	1 (8.3) 1	0	2 (13.3) 2
Other TEAEs										
Palpitations	1 (33.3) 1	0	2 (18.2) 2	0	0	1 (6.7) 1	0	0	2 (18.2) 2	1 (6.7) 1
Tinnitus	0	0	0	0	1 (9.1) 1	0	1 (9.1) 1	0	1 (9.1) 2	0
Gastroenteritis	0	0	1 (9.1) 1	0	0	0	0	0	1 (9.1) 1	0
ECG abnormal	0	0	0	0	0	1 (6.7) 1	0	0	0	1 (6.7) 1
Pelvic discomfort	0	0	0	1 (6.7) 1	0	0	0	0	0	1 (6.7) 1
Oropharyngeal pain	0	0	0	0	1 (9.1) 1	0	0	0	1 (9.1) 1	0
Ecchymosis	0	0	0	0	1 (9.1) 1	0	0	0	1 (9.1) 1	0

Notes

Percentages are based on all subjects in the Safety Analysis Set. Adverse events were classified into system organ class and preferred term using version 15.1 of MedDRA. Subjects were counted once per system organ class and once per preferred term per dose level/treatment group. Subjects were counted by the dose level/treatment group most recently taken when the event occurred.

Abbreviations: ECG, electrocardiogram; m, number of events; n, number of subjects experiencing the event; TEAE, treatment‐emergent adverse event.

Five subjects, all randomized to lisdexamfetamine, discontinued due to TEAEs, including two Japanese subjects (one due to increased blood pressure [on Day 4 after receiving the single dose of 20 mg on Day 1] and one due to dizziness [Day 17, while receiving 70 mg/d]) and three Caucasian subjects (one due to ECG abnormality: nonspecific *T*‐wave changes in anterior leads from which the subject recovered fully [Day 10, while receiving 50 mg/d], one due to dizziness [Day 12, while receiving 50 mg/d], and one due to anxiety [Day 14, while receiving 70 mg/d]). AE profiles were generally similar across the two ethnic groups. No unexpected or novel TEAEs were observed.

The postdose means for SBP, DBP, and pulse rate during both treatment periods were higher in the lisdexamfetamine groups than in the placebo groups, consistent with the known safety profile. Postsingle dose means for SBP, DBP, and pulse rate were generally higher for Japanese subjects than for Caucasians (Table 5), with greater magnitude of change among Japanese subjects. However, this pattern was also observed in subjects who received placebo during the single‐dose period. At 48 hours after receiving the single dose, SBP, DBP, and pulse rate were similar across all four groups of subjects, regardless of treatment or ethnicity. For subjects receiving multiple‐dose lisdexamfetamine, postdose means for SBP, DBP, and pulse rate were of similar magnitude in Japanese and Caucasian subjects. For subjects receiving placebo during the multiple‐dose period, postdose means for SBP, DBP, and pulse rate were generally higher in Japanese than Caucasian subjects (Table 6). When considering 4‐hour post multiple‐dose values, mean SBP, mean DBP, and mean pulse rate were higher in the lisdexamfetamine groups than in the placebo groups after Day 9, with generally greater changes from baseline seen in the lisdexamfetamine groups. Occasionally, values for SBP, DBP, and pulse rate were observed as being PCI, mostly in the lisdexamfetamine groups (Table 7). However, these PCI values occurred with similar frequency in both ethnic groups.

**Table 5:** Mean SBP, DBP, and pulse rate by ethnic group and treatment during the single‐dose period

	Placebo				Single lisdexamfetamine dose			
	Japanese (n = 3)		Caucasian (n = 3)		Japanese (n = 12)		Caucasian (n = 16)	
	Mean (SD)	Mean (SD) change from BL	Mean (SD)	Mean (SD) change from BL	Mean (SD)	Mean (SD) change from BL	Mean (SD)	Mean (SD) change from BL
Pulse rate (bpm)								
Screening	64.44 (7.76)		66.11 (11.66)		66.11 (8.35)		62.96 (7.76)	
Day −1	64.44 (11.97)		70.22 (10.89)		65.69 (7.25)		64.17 (8.67)	
Day 1 predose	69.44 (14.07)		63.44 (12.17)		64.56 (8.51)		64.52 (12.36)	
8 h postdose	71.67 (6.03)	2.22 (8.22)	67.67 (12.34)	4.22 (2.99)	76.97 (12.08)	12.42 (6.33)	70.54 (12.64)	6.02 (10.11)
12 h postdose	71.33 (12.12)	1.89 (5.87)	63.33 (9.60)	−0.11 (5.09)	78.94 (16.41)	14.39 (12.50)	67.42 (9.52)	2.90 (7.95)
24 h postdose	64.33 (6.89)	−5.11 (7.18)	60.56 (3.10)	−2.89 (9.86)	69.33 (14.69)	4.78 (9.77)	63.87 (8.04)	−1.20 (8.70)
48 h postdose	67.78 (4.86)	−1.67 (9.21)	62.22 (6.38)	−1.22 (6.19)	64.39 (8.78)	−0.17 (5.57)	63.27 (10.01)	−1.80 (8.93)
SBP (mm Hg)								
Screening	125.11 (10.34)		120.00 (1.20)		118.83 (8.11)		118.54 (8.62)	
Day −1	118.67 (12.66)		119.67 (7.51)		120.67 (7.91)		118.00 (7.51)	
Day 1 predose	124.33 (13.98)		114.67 (8.37)		115.81 (8.40)		112.90 (11.88)	
8 h postdose	113.44 (17.90)	−10.89 (5.85)	111.11 (7.20)	−3.56 (3.37)	119.00 (7.91)	3.19 (5.44)	113.10 (7.19)	0.21 (11.36)
12 h postdose	113.00 (18.19)	−11.33 (7.69)	111.33 (3.51)	−3.33 (5.78)	120.94 (15.13)	5.14 (10.68)	112.29 (6.99)	−0.60 (12.46)
24 h postdose	118.33 (14.88)	−6.00 (3.46)	109.44 (8.18)	−5.22 (4.91)	117.11 (14.48)	1.31 (10.74)	111.42 (7.37)	−1.27 (8.83)
48 h postdose	114.78 (12.36)	−9.56 (4.22)	109.78 (4.22)	−4.89 (6.50)	111.56 (10.28)	−4.25 (8.00)	111.22 (6.54)	−1.47 (9.46)
DBP (mm Hg)								
Screening	74.67 (4.63)		75.00 (2.65)		76.11 (6.13)		73.58 (6.42)	
Day −1	71.00 (8.97)		75.00 (2.91)		74.61 (3.86)		72.44 (6.89)	
Day 1 predose	73.56 (7.43)		69.33 (4.49)		71.50 (7.14)		69.06 (6.36)	
8 h postdose	64.11 (7.60)	−9.44 (5.55)	62.89 (1.39)	−6.44 (3.10)	68.17 (8.77)	−3.33 (3.24)	66.33 (4.28)	−2.73 (6.14)
12 h postdose	67.89 (8.22)	−5.67 (7.62)	65.78 (2.17)	−3.56 (5.39)	69.19 (9.26)	−2.31 (4.38)	67.50 (5.57)	−1.56 (7.08)
24 h postdose	71.11 (8.80)	−2.44 (6.20)	64.89 (3.37)	−4.44 (6.38)	71.92 (7.79)	0.42 (4.73)	69.62 (4.77)	0.80 (5.41)
48 h postdose	69.11 (3.86)	−4.44 (4.34)	69.44 (0.51)	0.11 (4.91)	67.42 (6.81)	−4.08 (5.06)	69.33 (6.07)	0.51 (5.49)

Note

Baseline for predose measurements is the first available measurement from Day 1 predose, Day −1, or screening (missing if all three missing). Baseline for postdose measurements is the predose measurement from the same sequence (missing if that predose measurement is missing). Values are reported for a range of all the evaluated time points.

Abbreviations: BL, baseline; bpm, beats per minute; DBP, diastolic blood pressure; SBP, systolic blood pressure; SD, standard deviation.

**Table 6:** SBP, DBP, and pulse rate by ethnic group and treatment during the multiple‐dose period

Parameter	Placebo				Multiple lisdexamfetamine dose^a^			
	Japanese (n = 3)		Caucasian (n = 3)		Japanese (n = 12)		Caucasian (n = 16)	
	Mean (SD)	Mean (SD) change from BL	Mean (SD)	Mean (SD) change from BL	Mean (SD)	Mean (SD) change from BL	Mean (SD)	Mean (SD) change from BL
Pulse rate (bpm)								
Screening	64.44 (7.76)		66.11 (11.66)		66.11 (8.35)		62.96 (7.76)	
Day −1	64.44 (11.97)		70.22 (10.89)		65.69 (7.25)		64.17 (8.67)	
Day 1 predose	69.44 (14.07)		63.44 (12.17)		64.56 (8.51)		64.52 (12.36)	
Day 4 predose	63.22 (10.57)	−6.22 (4.03)	58.89 (5.87)	−4.56 (6.38)	64.06 (9.19)	0.06 (4.44)	63.60 (10.21)	−1.47 (11.05)
Day 4, 4 h postdose	67.22 (10.68)	4.00 (1.45)	62.56 (9.10)	3.67 (3.76)	75.39 (7.25)	11.33 (7.58)	69.38 (11.63)	5.78 (9.20)
Day 9 predose	66.33 (11.78)	−3.11 (8.04)	60.33 (5.36)	−3.11 (7.17)	67.67 (8.54)	3.67 (5.41)	66.42 (8.80)	1.36 (8.87)
Day 9, 4 h postdose	64.78 (11.24)	−1.56 (0.84)	62.56 (6.74)	2.22 (4.60)	81.30 (5.88)	13.64 (7.06)	82.11 (18.31)	15.69 (15.82)
Day 14 predose	67.22 (6.92)	−2.22 (8.34)	64.11 (8.22)	0.67 (7.37)	69.27 (8.38)	5.27 (7.30)	66.97 (8.57)	2.17 (6.48)
Day 14, 4 h postdose	69.22 (0.84)	2.00 (7.17)	66.22 (7.20)	2.11 (4.17)	78.97 (5.85)	9.70 (8.35)	82.61 (15.44)	15.64 (11.84)
Day 18 predose	67.00 (10.02)	−0.22 (3.56)	61.44 (8.04)	−2.67 (4.26)	72.60 (7.14)	4.20 (3.95)	71.82 (10.29)	4.79 (8.69)
Day 18, 4 h postdose	62.44 (8.53)	−4.78 (6.16)	61.89 (10.19)	−2.22 (5.42)	79.80 (6.08)	11.40 (8.90)	74.94 (16.86)	7.91 (14.43)
SBP (mm Hg)								
Screening	125.11 (10.34)		120.00 (1.20)		118.83 (8.11)		118.54 (8.62)	
Day −1	118.67 (12.66)		119.67 (7.51)		120.67 (7.91)		118.00 (7.51)	
Day 1 predose	124.33 (13.98)		114.67 (8.37)		115.81 (8.40)		112.90 (11.88)	
Day 4 predose	115.44 (7.40)	−8.89 (6.59)	112.67 (5.81)	−2.00 (6.89)	110.79 (6.97)	−4.55 (6.07)	114.60 (6.47)	1.91 (10.34)
Day 4, 4 h postdose	123.22 (10.54)	7.78 (3.37)	110.11 (6.68)	−2.56 (4.54)	118.73 (9.31)	7.94 (7.44)	116.60 (7.67)	2.00 (6.58)
Day 9 predose	112.56 (6.26)	−11.78 (10.01)	110.22 (3.01)	−4.44 (5.87)	109.91 (8.53)	−5.42 (4.70)	112.24 (6.30)	−0.44 (11.66)
Day 9, 4 h postdose	113.89 (6.62)	1.33 (5.29)	109.56 (6.94)	−0.67 (4.06)	124.39 (10.35)	14.48 (7.96)	122.18 (6.38)	9.93 (6.83)
Day 14 predose	113.56 (2.22)	−10.78 (11.77)	110.22 (0.96)	−4.44 (7.41)	110.33 (9.19)	−5.00 (9.74)	111.36 (6.69)	−1.64 (17.24)
Day 14, 4 h postdose	108.56 (7.18)	−5.00 (4.98)	110.11 (0.69)	−0.11 (0.69)	123.79 (8.39)	13.45 (12.33)	123.44 (5.47)	12.08 (7.30)
Day 18 predose	111.56 (6.20)	−2.00 (5.69)	107.56 (1.07)	−2.67 (1.16)	111.30 (6.09)	2.63 (8.78)	111.30 (6.80)	−1.27 (7.86)
Day 18, 4 h postdose	110.56 (4.03)	−3.00 (6.25)	113.22 (3.95)	3.00 (3.53)	122.83 (11.20)	14.17 (11.89)	122.79 (6.02)	10.21 (5.75)
DBP (mm Hg)								
Screening	74.67 (4.63)		75.00 (2.65)		76.11 (6.13)		73.58 (6.42)	
Day −1	71.00 (8.97)		75.00 (2.91)		74.61 (3.86)		72.44 (6.89)	
Day 1 predose	73.56 (7.43)		69.33 (4.49)		71.50 (7.14)		69.06 (6.36)	
Day 4 predose	70.44 (6.50)	−3.11 (1.02)	69.11 (1.71)	−0.22 (6.00)	68.64 (6.99)	−2.67 (6.68)	68.87 (5.68)	0.04 (7.36)
Day 4, 4 h postdose	73.00 (3.18)	2.56 (3.60)	65.33 (2.03)	−3.78 (0.51)	72.97 (7.72)	4.33 (7.08)	68.64 (5.57)	−0.22 (3.83)
Day 9 predose	68.00 (3.76)	−5.56 (3.72)	67.00 (2.91)	−2.33 (1.67)	69.09 (6.10)	−2.21 (4.21)	69.02 (4.62)	0.20 (6.11)
Day 9, 4 h postdose	67.11 (4.34)	−0.89 (2.17)	64.56 (2.72)	−2.44 (5.58)	75.18 (6.33)	6.09 (2.99)	74.22 (6.65)	5.20 (4.15)
Day 14 predose	69.33 (3.71)	−4.22 (7.53)	69.78 (4.68)	0.44 (5.27)	69.94 (7.61)	−1.36 (6.48)	71.58 (5.82)	2.69 (8.85)
Day 14, 4 h postdose	66.22 (3.91)	−3.11 (6.94)	63.56 (2.01)	−6.22 (2.67)	74.73 (5.24)	4.79 (7.50)	76.06 (6.96)	4.47 (5.34)
Day 18 predose	66.22 (6.38)	−3.11 (4.53)	67.89 (2.78)	−1.89 (2.04)	69.63 (5.41)	1.20 (5.79)	71.42 (3.51)	−0.79 (6.05)
Day 18, 4 h postdose	66.67 (1.20)	−2.67 (3.38)	69.22 (2.84)	−0.56 (4.22)	77.63 (6.66)	9.20 (7.17)	78.06 (5.08)	5.85 (3.17)

Note

Baseline for predose measurements is the first available measurement from Day 1 predose, Day −1, or screening (missing if all three missing). Baseline for postdose measurements is the predose measurement from the same sequence (missing if that predose measurement is missing). Values are reported for a range of all the evaluated time points.

Abbreviations: BL, baseline; bpm, beats per minute; DBP, diastolic blood pressure; SBP, systolic blood pressure; SD, standard deviation.

a Multiple lisdexamfetamine doses were given per the dosing schedule described above.

**Table 7:** The number of subjects with pulse, SBP, or DBP values that were considered potentially clinically important by ethnic group and treatment, n (%)

Vital sign	Placebo		Lisdexamfetamine	
	Japanese (n = 3)	Caucasian (n = 3)	Japanese (n = 12)	Caucasian (n = 16)
Pulse (bpm)				
≤50	0 (0)	1 (33.3)	1 (8.3)	4 (25.0)
≥100	0 (0)	0 (0)	4 (33.3)	5 (31.3)
SBP (mm Hg)				
<100	1 (33.3)	1 (33.3)	3 (25.0)	6 (37.5)
>140	1 (33.3)	0 (0)	4 (33.3)	3 (18.8)
DBP (mm Hg)				
<60	2 (66.7)	2 (66.7)	5 (41.7)	7 (43.8)
>90	0 (0)	0 (0)	2 (16.7)	1 (6.3)

Abbreviations: bpm, beats per minute; DBP, diastolic blood pressure; SBP, systolic blood pressure.

No clinically meaningful changes or trends were noted in clinical laboratory data or vital signs, other than the one ECG discontinuation noted above. Caucasian subjects in the lisdexamfetamine group experienced a greater mean weight loss than Japanese subjects (−1.45 kg vs −0.50 kg, respectively). This interethnic difference was also evident in the placebo group. However, Japanese subjects experienced a gain from baseline weight: −0.53 kg and +0.70 kg from baseline for Caucasian and Japanese subjects, respectively. There were no positive responses to any of the C‐SSRS questions concerning suicidal behavior, suicidal ideation, or suicide attempts at any time point during the study.

## 4 DISCUSSION

In this study, single (20 mg) and multiple increasing doses (20, 50, and 70 mg/d) of lisdexamfetamine (a prodrug stimulant) were administered to healthy subjects of Japanese and Caucasian descent. Lisdexamfetamine and the therapeutically active d *‐*amphetamine exhibited plasma concentration‐time curves similar in shape for Japanese and Caucasian subjects in the single‐ and multiple‐dose periods. AE profiles were similar across the two ethnic groups, and all TEAEs were mild or moderate in severity.

The *C*_max_ for d *‐*amphetamine reported here was approximately 34% higher in Japanese than Caucasian subjects during the single‐dose period, and at each lisdexamfetamine dose level in the multiple‐dose period (range approximately 16%‐24% higher for d *‐*amphetamine), resulting in higher mean AUCs in Japanese than Caucasian subjects (range approximately 11%‐15% higher for d *‐*amphetamine during the multiple‐dosing period). The observed differences in *C*_max_ and AUC for lisdexamfetamine and d *‐*amphetamine could potentially be due to bodyweight differences between Japanese and Caucasian subjects. On average in the lisdexamfetamine groups, Japanese subjects were approximately 17% lighter than Caucasian subjects. Weight‐corrected means for CL/F were similar in Japanese and Caucasian subjects.

The AUC_τ_ and *C*_max_ for d *‐*amphetamine behaved in a linear, dose‐proportional manner across the dose range studied for both Japanese and Caucasian subjects, reinforcing previous findings.^4^, ^5^ There were no unexpected accumulations of d *‐*amphetamine in Japanese or Caucasian subjects.

In the study reported here, lisdexamfetamine was absorbed, metabolized, and excreted in a similar manner in Japanese and Caucasian subjects. Greater mean exposure was observed in Japanese subjects, potentially also due to differences in bodyweight. *T*_max_ and *t*_½_ of both lisdexamfetamine and d *‐*amphetamine were similar across both groups and in accordance with a previous study in healthy adults.^5^

With regard to safety, single and multiple doses of lisdexamfetamine were generally well tolerated in the current study, with both Japanese and Caucasian subjects experiencing similar, generally mild TEAEs that were resolved without therapeutic intervention. TEAEs were consistent with the established safety profile of lisdexamfetamine reported in phase 3 clinical trials.^16^, ^17^, ^27^

In the current study, subjects from both ethnic groups had changes in SBP and DBP following exposure to lisdexamfetamine. However, small mean changes in blood pressure and accompanying pulse rates have been previously reported in a number of other clinical studies of lisdexamfetamine.^16^, ^17^, ^28^, ^29^, ^30^ The observed changes in SBP and DBP are consistent with the safety profile of lisdexamfetamine and are largely anticipated due to the sympathomimetic effects of lisdexamfetamine. It is important to note that mean SBP and DBP for Japanese subjects reported here were similar in magnitude to those for Caucasian subjects despite the slightly higher exposure to d *‐*amphetamine observed in Japanese subjects.

When the dose of lisdexamfetamine was increased to 70 mg/d, the predose pulse rate appeared to be higher compared with the value at screening. This is not unexpected and has also been seen with other products containing d *‐*amphetamine in Western populations. Lisdexamfetamine 70 mg once daily generally is associated with increased pulse rate versus baseline over the course of the day.^24^ This has not previously been associated with a safety risk when used according to label instructions in Western populations, but experience in a Japanese population is more limited, which may have some clinical significance. One subject discontinued lisdexamfetamine treatment due to ECG changes. Discontinuation due to changes in ECG parameters following lisdexamfetamine treatment has been reported in a short‐term, randomized, double‐blind trial of patients aged 6‐12.^31^ However, it has been suggested that these discontinuations were impacted by variations in ECG interpretation.^32^ Furthermore, an open‐label prospective study of cardiovascular health in adults found no clinically meaningful changes with regards to cardiac function, physiology, or structure.^33^ Therefore, the ECG changes exhibited by a single subject in this study were not considered a significant signal that departed from the known safety profile of lisdexamfetamine.

There were no serious or severe TEAEs and no new or unexpected safety findings in the study. Generally, no notable differences were observed between Japanese and Caucasian subjects.

These results must be interpreted with caution due to the small sample size and the population studied (healthy adults) as it may not accurately reflect the general population or the full spectrum of subjects with ADHD, which includes children and adolescents. An additional limitation is the short follow‐up period of this study.

Overall, the data from this phase 1 study suggest that lisdexamfetamine may be administered to Japanese subjects at the same dose range as that used in Caucasian subjects, without any additional concerns relating to increased drug exposure or safety. However, studies in larger populations of Japanese subjects with ADHD will further elucidate the safety and pharmacokinetic profile of lisdexamfetamine in this population.

## CONFLICTS OF INTEREST

JE is a former employee of Shire. PM and MC are employees of Shire and hold stock and/or stock options in Shire. YM is an employee of Shionogi & Co., Ltd.

## AUTHOR CONTRIBUTIONS

All individuals included as authors of papers contributed substantially to the scientific process leading up to the writing of the paper.

JE, MC, PM, and YM conceptualized and designed the study; JE, MC, PM, and YM involved in acquisition and analysis of data; JE, MC, PM, and YM: drafted the manuscript and figures.

All authors take final responsibility for the decision to submit for publication and have approved the final version of the paper. All authors had full access to all of the data in the study and take responsibility for the integrity of the data and the accuracy of the data analysis. The authors are entirely responsible for the scientific content of the paper.

## DATA REPOSITORY

The datasets, including the redacted study protocol, redacted statistical analysis plan, and individual participant's data behind the results reported in this article, will be available 3 months after the submission of a request, to researchers who provide a methodologically sound proposal after de‐identification in compliance with applicable privacy laws, data protection, and requirements for consent and anonymization.

Data listings are not publicly available for this study because consent for all data directly associated with the results to be made available in a permanent, publicly accessible data archive, or repository was not obtained in the patient consent forms.

## APPROVAL OF THE RESEARCH PROTOCOL BY AN INSTITUTIONAL REVIEWER BOARD

The protocol for this research project has been approved by a suitably constituted ethics committee of the institution and it confirms to the Declaration of Helsinki. Approval was provided by Alpha IRB, San Clemente, CA.

## INFORMED CONSENT

All informed consent was obtained from the subjects and/or guardians.

## REGISTRY AND REGISTRATION NUMBER OF THE TRIAL

Not applicable.

## ANIMAL STUDIES

Not applicable.
